# Socioeconomic Inequalities in Quality of Life in Iranian Children and Adolescents: The Weight Disorder Survey of the CASPIAN-IV Study

**Published:** 2019-07-13

**Authors:** Silva Hovsepian, Mostafa Qorbani, Mojgan Asadi, Motahareh Hatami, Mohammad Esmaeil Motlagh, Armita Mahdavi-Gorabi, Mehdi Noroozi, Roya Kelishadi

**Affiliations:** ^1^Pediatrics Department, Child Growth and Development Research Center, Research Institute for Primordial Prevention of Non-Communicable Disease, Isfahan University of Medical Sciences, Isfahan, Iran; ^2^Non-communicable Diseases Research Center, Alborz University of Medical Sciences, Karaj, Iran; ^3^Endocrinology & Metabolism Research Center, Endocrinology and Metabolism Clinical Sciences Institute, Tehran University of Medical Sciences, Tehran, Iran; ^4^Osteoporosis Research Center, Endocrinology and Metabolism Clinical Sciences Institute, Tehran University of Medical Sciences, Tehran, Iran; ^5^Chronic Diseases Research Center, Endocrinology and Metabolism Population Sciences Institute, Tehran University of Medical Sciences, Tehran, Iran; ^6^Department of Pediatrics, Ahvaz Jundishapur University of Medical Sciences, Ahvaz, Iran .; ^7^Department of Basic and Clinical Research, Tehran Heart Center, Tehran University of Medical Sciences, Tehran, Iran; ^8^Social Determinants of Health Research Center, University of Social Welfare and Rehabilitation Sciences, Tehran, Iran

**Keywords:** Quality of life, Child, Socioeconomic factors, Adolescent, Iran

## Abstract

**Background:** We aimed to determine the association between socioeconomic status (SES) and health-related quality of life (HRQOL) in a representative sample of Iranian children and adolescents.

**Study design:** A cross-sectional study.

**Methods:** In this nationwide school-based study, 6-18-yr-old students were selected via multistage cluster random sampling method from 30 provinces of Iran in 2011-2012. SES of each participant was determined using the categories of the Progress International Reading Literacy Study (PIRLS) for Iran and the Principle Component Analysis (PCA) method. The students’ HRQL was evaluated using the Persian version of the Pediatric Quality of Life Inventory (PedsQL™ 4.0TM 4.0) Generic Core Scales. The level of physical activity was evaluated using the physical activity questionnaire for adolescents. The association between SES and HRQL was evaluated using multiple linear regression analysis.

**Results:** Overall, 23043 students were enrolled. The mean of total PedsQL™ score, school function, and psychosocial subscales was significantly different between different categories of SES (*P*<0.001). The differences in total population and among girls were between low and middle categories and low and high categories, but in boys, the difference was between low and high categories. There was a significant association between SES and school functioning, psychosocial function, and total score of HRQOL. Moderate and high SES had higher score compared to low SES group [β=1.0(0.5-1.6) for Moderate SES/Low SES and β=1.6(1.0-2.2) for High SES/Low SES].

**Conclusion:** SES is in positive association with HRQOL of Iranian schoolchildren, mainly due to its impact on school function. HRQOL could be improved by elimination of the socio-economic inequalities especially in the field of school function.

## Introduction

Health-related quality of life (HRQoL) is an influential health outcome measure. It is also defined as self-perceived health which includes individual's perceived physical, mental, emotional health, and social wellbeing. It is a multidimensional concept which focuses on all aspects of health concurrently ^1,2^.

HRQOL is a valuable indicator for decision making in clinical practice and administration of relevant public health policy. It has the capacity to describe personal health status in both general representative and disease-specific groups ^3^. It has gained increasing attention in pediatric health care and is considered as a new concept in this field.

The evaluation of pediatric HRQOL is considered a challenging issue, due to different developmental phases of which, its measurement is not fully utilized in this group of population. Several studies have investigated the association of various factors and HRQOL in children and adolescents. Accordingly, gender, age, demographic factors, and socioeconomic status (SES) were reported as potential variables related to HRQOL. Evidence indicates lower HRQOL in girls, older children, and families with lower SES-^7^. In Germany, higher level of quality of life was reported in higher SES composite score. One of the above-mentioned determinants of HRQOL is SES. Different variables are inclusive in the measurement of SES such as family income, parents’ education, and occupational prestige. SES of family may affect children’s life opportunities in various fields including health and quality of life ^8^.

The impact of SES on health outcomes in adults is well established in previous researches using different measures for SES ^9,10^. There are few types of research among pediatric population, and the results are inconsistent ^11-14^. In addition, there are few population-based studies in children and adolescents in this field. Chronic diseases are less common in childhood period than adulthood, evaluation of HRQOL in a population-based study would be helpful for proper identification of children with low HRQOL and its related determinants neglected from the perception of social and health care system. In addition, pediatric age is a critical period for implementation of targeted lifestyle and behavioral intervention in preventive medicine.

We aimed to determine the association between SES and HRQOL in a representative sample of Iranian children and adolescents. The outcomes of this study would be applicable to health care policies.

## Methods

This cross-sectional study was designed as a sub-study of the Childhood Adolescence Surveillance and Prevention of Adult Non-Communicable Disease (CASPIAN- IV) study entitled the Weight Disorder Survey of the CASPIAN-IV study. The Weight Disorder Survey (WDS) of the CASPIAN IV nationwide school-based study was conducted in 2011-2012 in 30 provinces of Iran among students aged 6-18 years. Details and methodology of both studies (the CASPIAN-IV study and the WDS) were previously published ^15,16^.

Here in, the methods of the current study are explained briefly. In the WDS, school students, aged 6-18 yr, were selected via multistage cluster random sampling method from rural and urban areas of 30 provinces of Iran. The schools were selected by random sampling method from the list of schools provided by the information bank of the Ministry of Education. Desired number of samples was obtained using cluster sampling in each province with equal cluster sizes. In each province, 83 clusters and in each cluster 10 students were selected randomly. In the WDS, the sample size was calculated according to the following formula:

In this formula, type I, prevalence, and precision were considered 5%, 50%, and 8.5% respectively. For two sexes, 31 provinces, and three age groups (7-10, 11-14, and 15-18 yr old), total sample sizes were approximately calculated, 25000 students. Data of one province were not available ^16^.

$$n=\frac{z^{2}{}_{{}_{1-\frac{\alpha }{2}}}P(1-P)}{d^{2}}$$

The study included schoolchildren with Iranian citizenship aged 6-18 yr who accepted to participate in the study. Schoolchildren with a medical history of any chronic disorder, physical disability, or prolonged use of any medication were excluded from the study.

The Ethics Committee of Isfahan University of Medical Sciences approved the protocol of the study (ID number: 188092). After describing the goals and methods of the study, oral assent and written informed consent were obtained from the selected students and their parents.

Using the validated questionnaires of the CASPIAN IV study, demographic and family-based characteristics of the students were recorded by trained interviewers. The students and their parents completed two sets of questionnaires. The students’ questionnaire was obtained from the Persian version of the World Health Organization-Global School-Based Student Health Survey (WHO-GSHS). It had a content validity of 0.75 and reliability of 0.94 ^17^.

Anthropometric measurements including weight and height of the students were conducted by trained healthcare stuff according to standard protocols using calibrated instruments. BMI of each participant was calculated as weight (kg) divided by height squared (m^2^). BMI was classified in four categories as follows based on WHO growth curve: underweight (less than or equal to 5th percentile), normal weight (between 5th and 85th percentiles), overweight (between 85th and 95th percentiles), and obese (equal to or more than 95th percentile)^15^. Level of physical activity was evaluated using the physical activity questionnaire for adolescents (PAQ-A) which is the validated and modified version of the physical activity questionnaire for children (PAQ-C). It is a self-administrated questionnaire which records the subjects’ sports or activities during their spare time, physical education period, after school, in the evenings, and on weekends during a 7-day period. It is classified as low and high physical activity level as the PAQ-A score was 1-1.9 and 2-5, respectively^18^.

Screen time (ST) was determined by asking the average time (hours per day) on weekdays and weekends the students spent watching TV, electronic games, or leisure time computer use. Level of ST was classified low (<2 h per day) and high (>2 h per day)^15^.

### Socioeconomic status (SES)

SES categories were determined according to the methods and variables of the Progress in International Reading Literacy Study (PIRLS).

Baseline information for calculating the SES of the participants was obtained from the parents’ questionnaire. The SES was determined by the Principle Component Analysis (PCA) method based on variables including parents’ education, parents’ occupation, family possessions of private car and personal computer, school type of children (public/private), and type of home (rental/private). The weighted averages of the variables according to the PCA method were summarized as one main component, defined as the SES score. Obtained scores were categorized into tertiles. The first, second, and third tertiles were classified as low, middle, and high SES, respectively ^15^.

### Assessment of HRQL

The Pediatric Quality of Life Inventory (PedsQL™ 4.0TM 4.0) is a well-established tool with multidimensional constructs containing 23 items in four subscales including physical, emotional, social, and school functions. Physical function contains eight items, and the other three items were defined as psychosocial score have five items. The composite of the physical and psychosocial scores were defined as total score. The scale is scored from 0-100; higher scores reflected better HRQOL^19^.

The Persian version of the PedsQL™ 4.0TM 4.0 scales the validity and reliability of previously confirmed was used for assessment of the students’ HRQL. The internal consistency of this questionnaire was 0.95 and 0.91 for parent-proxy and child-self reports, respectively. The Cronbach's alpha for the total scale score was 0.73 and 0.9 for child-self and parent-proxy reports, respectively ^19^.

### Statistical analysis

The data were analyzed using the STATA software version 10.0 (STATA Corp, College Station, Tex.). Categorical and continuous variables are presented as number (%) and mean (standard deviation, SD), respectively. The categorical variables were analyzed by the Pearson Chi-square test. Comparison of means of total PedsQL™ and its subscales across SES were investigated by analysis of variance (ANOVA). Linear regression was performed to examine the association of SES with total PedsQL™ and its subscales in different models applied for adjusting potential confounders. Model I was a crude model; Model II was adjusted for age, sex, and living area, and in Model III, the additional adjustment was done for PA, ST, and BMI. Sampling method (cluster sampling) was considered in all statistical analyses. *P*-value<0.05 was considered as statistically significant.

## Results

Overall, 23043 students (50.8% boys) with mean (SD) age of 12.55±3.41 yr out of 25000 invited students completed the survey (participation rate: 92.17%); 73.4% of the participants were from urban regions.

General characteristics, total PedsQL™, and its subscales and SES of the participants according to gender are presented in Table 1. Mean of total PedsQL™ was 81.7+13.3; for boys, it was 9.87±14.41 and for girls 8.36±12.27. The mean of total PedsQL™ and its subscales were significantly higher in boys compared to girls. Social subscale had higher mean. Mean (SD) of total PedsQL™ and its subscales according to SES are presented in Table 2. Mean of total PedsQL™ score, school function, and psychosocial subscales were significantly different between different categories of SES (*P*<0.001). The differences in total population and among the girls were between low and middle categories and low and high categories, but in the boys, the difference was between low and high categories.

**Table 1:** General characteristics, total PedsQL™ and its subscales and socioeconomic status according to gender: the weight disorders survey of the CASPIAN IV

**Continuous variables**	**Total**		**Boy**		**Girls**		***P*** **value**
	**Mean**	**SD**	**Mean**	**SD**	**Mean**	**SD**	
Age (yr)	12.5	3.4	12.3	3.3	12.7	3.2	0.001
Body mass index (kg/m^2^)	18.7	4.4	18.6	4.3	18.9	4.4	0.001
Quality of life components							
Physical	84.2	14.7	84.5	14.4	83.9	14.9	0.002
School	78.6	14.5	82.2	13.1	75.0	15.1	0.001
Emotional	78.2	19.4	81.0	18.2	75.4	20.2	0.001
Social	90.0	14.2	89.9	14.4	90.1	14.1	0.640
Psychosocial	81.2	14.1	83.6	12.9	78.8	14.8	0.001
Total	81.7	13.6	83.5	12.3	79.8	14.5	0.001
**Categorical variables**	**Number**	**Percent**	**Number**	**Percent**	**Number**	**Percent**	***P*** **value**
Living area							0.001
Urban	14218	73.0	6952	74.9	7266	74.9	
Rural	5267	27.0	2431	25.1	2836	29.0	
Socioeconomic status							0.500
Low	7696	33.4	7650	33.2	7765	33.7	
Middle	7650	33.2	7650	32.6	7719	33.5	
High	7696	33.4	7880	34.2	7650	32.7	

**Table 2:** Mean (SD) of total PedsQL™ and its subscales according to socioeconomic status: the weight disorders survey of the CASPIAN IV

**Socioeconomic status**	**Low**		**Moderate**		**High**		***P*** **value**
	**Mean**	**SD**	**Mean**	**SD**	**Mean**	**SD**	
Girls							
Physical	84.0	14.7	84.0	14.7	83.6	15.3	0.390
Emotional	76.1	19.9	75.5	19.9	75.2	20.5	0.340
Social	90.3	13.8	90.2	13.6	89.8	14.5	0.410
School function	73.6	16.2	75.3	14.7	75.8	14.3	0.001
Psychosocial	77.6	16.3	79.0	14.5	79.6	14.0	0.001
Total	78.6	16.0	80.0	14.1	80.6	13.5	0.001
Boys							
Physical	84.7	14.7	84.2	14.4	84.3	14.4	0.610
Emotional	81.0	18.5	80.7	18.3	81.0	18.1	0.780
Social	90.1	14.2	89.6	14.1	90.2	14.7	0.240
School function	81.4	13.8	82.3	12.7	82.8	12.9	0.001
Psychosocial	82.6	14.0	83.6	12.5	84.3	12.5	0.001
Total	82.6	13.6	83.5	11.9	84.2	11.8	0.001
Both genders							
Physical	84.3	14.7	84.2	14.5	84.0	14.8	0.490
Emotional	76.1	19.9	78.1	19.3	78.2	19.6	0.540
Social	90.2	14.0	89.9	13.8	90.0	14.6	0.650
School function	77.5	15.5	82.3	12.7	79.4	14.0	0.001
Psychosocial	80.2	15.3	81.2	13.7	82.0	13.5	0.001
Total	80.6	14.9	81.7	13.2	82.4	12.8	0.001

Association of SES with total PedsQL™ and its subscales in linear regression models are presented in Table 3. In multivariate model (model III), there was a significant association between SES and school functioning, psychosocial function, and total score of HRQOL; moderate and high SES had higher score compared to low SES group (*P*<0.05).

**Table 3:** Association of socioeconomic status with total PedsQL™ and its subscales: the weight disorders survey of the CASPIAN IV

**SES category**	**Physical**	**Emotional**	**Social**	**School** **function**	**Psychosocial** **function**	**Total**
	**B** ^d^ **(95% CI)**	**B** ^d^ **(95% CI)**	**B** ^d^ **(95% CI)**	**B** ^d^ **(95% CI)**	**B** ^d^ **(95% CI)**	**B** ^d^ **(95% CI)**
**Moderate SES/ Low SES**						
Model I ^a^	-0.2 (-0.8, 0.4)	-0.4 (-1.3, 0.4)	-0.2 (-0.9, 0.3)	1.2 (0.6, 1.4)	1.0 (0.5, 1.6)	1.0 (0.5, 1.6)
Model II^b^	-0.1 (-0.8, 0.4)	-0.3 (-1.2, 0.4)	-0.1 (-0.8, 0.4)	1.3 (0.7, 1.9)	1.1 (0.5, 1.7)	1.1 (0.5, 1.7)
Model III ^c^	-0.1 (-0.8, 0.4)	-0.4 (-1.3, 0.4)	-0.1 (-0.7, 0.5)	1.2 (0.6, 1.8)	1.0 (0.5, 1.6)	1.0 (0.5, 1.6)
**High SES/ Low SES**						
Model I ^a^	0.3 (-1.0, 0.3)	-0.3 (-1.2, 0.4)	-0.1 (-0.8, 0.4)	1.8 (1.2, 2.4)	1.8 (1.2, 2.4)	1.7 (1.2, 2.3)
Model II^b^	-0.3 (-0.9, 0.3)	-0.3 (-1.1, 0.6)	-0.0 (-0.7, 0.5)	1.8 (1.2, 2.4)	1.8 (1.2, 2.4)	1.8 (1.2, 2.4)
Model III ^c^	-0.4 (-1.1, 0.2)	-0.5 (-1.4, 0.3)	-0.0 (-0.7, 0.5)	1.6 (1.0, 2.3)	1.6 (1.0, 2.2)	1.6 (1.0, 2.2)

SES: socioeconomic status ^a^ Crude model ^b^ Adjusted for age, sex, and region ^c^ Adjusted for age, sex, region, and additionally adjusted for physical activity, body mass index, and screen time ^d^ Regression coefficient

The final model (model III) showed that with improving the SES level, the mean of total score of HRQOL increased to 1.6 (CI 95%:1.0, 2.2).

## Discussion

In this population-based study, we investigated the association between SES and HRQOL in Iranian children and adolescents. Mean score of total PedsQL™ and its school function and psychosocial subscales was significantly higher in high SES. There was significant association between SES and HRQOL mainly due to its impact on school functioning and psychosocial components.

The mean level of HRQOL in the study population was in its higher range both in total and in its all subscales, indicating that individuals with different SESs have a positive attitude towards their quality of life.

HRQOL is lower in girls than boys ^20-22^. In this study, the total score of HRQOL and its subscales except its social subscale were significantly lower in girls than boys. The results in this field were consistent with previous studies^20-22^.Likewise, the findings regarding the gender differences may be representative for genuine discrepancy between girls and boys. Some factors could explain the difference, such as biological differences in boys and girls regarding the age of puberty, brain development, and cultural differences with respect to gender role in the society such as provision of more opportunities for professional development of males in our community. More studies are required to investigate the most important factors related to the reported difference.

As mentioned earlier, there exist few studies evaluating the impact of SES or its inclusive variables on HRQOL among children and adolescents ^11-13,23,24^. In a national study in Australia, the differences in HRQOL of children were investigated with different SESs. Children with lower SES had lower level of HRQOL. Children with higher income families, parents with higher education, and individuals living in two-parent families had higher level of HRQOL^11^.

In Germany, the impact of SES measures was investigated on children’s self-reported HRQoL. Low SES is associated with lower HRQOL mainly with its physical health subscale. Children having better opportunities for social, cultural, and educational activities such as having a car in the family and/or a bedroom of their own experience different cultures during holidays, and those who have access to a personal computer have higher level of HRQOL ^12^.

In Brazil, the impact of socioeconomic factors was evaluated on the HRQOL of schoolchildren using PedsQL. Significant differences were reported for social, emotional, and psychosocial subscales and total scores of the study population in different SES. It did not report any significant association between SES and physical and school function of studied population ^13^.

In Germany, the association between SES and children’s health outcomes including quality of life was evaluated and reported higher level of quality of life in higher SES composite score ^25^.

In the current study, the mean level of total PedsQL, school function, and psychosocial subscales was significantly higher in middle SES and high SES compared to low SES in all studied population. The results in girls were similar to total population, but in boys, the difference was significant between high SES and low SES. Results of logistic regression analysis indicated a significant association between SES and school functioning, psychosocial function and total score of HRQOL between low SES and moderate SES and low SES and high SES.

There was not such an association with other subscales including emotional and social function, the main causes of observed association between SES and psychosocial function are possibly due to its effect on school function. School function is more influenced by different socio-economic conditions and its related factors such as family income, parents' education, or individuals' assessment of resources than other domains.

Since there are few studies in this field and the research methods of the above studies were not similar and were different regarding evaluation measures of HRQOL, we could not compare the findings with the studies in details. Thus, we compared overall findings and make a conclusion based on our study population characteristics.

The implication of this study was that if we could not eliminate the socioeconomic inequalities in our community, we could improve the level of HRQOL of children by improving educational programs of schools to reduce the impact of SES on school function of Iranian children and consequently improve public health in our community.

The main limitation of the current study was its cross-sectional design. For obtaining decisive results, longitudinal studies are more appropriate. In addition, the impact of different aspects of SES on HRQOL is varied ^11,13^. It is recommended to separately evaluate the influence of each inclusive variable of SES measurements such as parental education, parental occupation, family possessions of private car and personal computer, school type of children, and type of home on HRQOL in children. In addition, puberty is one of the important transition states in children, which may have a significant effect on HRQOL of studied population, as a limitation to the current study, we did not compare HRQOL in different age groups (pre-pubertal and pubertal).

In this study, we used the validated Persian version of PedsQL™ 4.0 ^26^. It is a generic instrument developed in the USA ^19^. PedsQL is a widely used instrument with proper psychometric properties which measures all core health dimensions recommended by WHO. It is a self-report and proxy-report measurement used for children aged 2 to 18 yr old, used in different settings including clinical trials, clinical practice, school health, as well as community-based settings^27,28^.

The large sample size of the studied population from 30 provinces of Iran is considered the strength of this study. However, some factors can make the interpretation of the results difficult. We used a single questionnaire for assessing SES in all provinces, whereas the minimum and maximum levels of family income are not similar in different cities. Thus, taking into account the deprivation rate, it is recommended to use questionnaires that are more appropriate.

## Conclusion

SES is in positive association with HRQOL of Iranian schoolchildren. The association was mainly due to its impact on school function. Our findings could be used as baseline information for health care policymakers to improve HRQOL of Iranian children by elimination of the socio-economic inequalities or their impact on school function of children by proper educational planning. It is also recommended to design studies to identify the causes of such inequalities and develop interventional study to prevent them.

## Acknowledgements

This nationwide survey was conducted in Iran with the cooperation of the Ministry of Health and Medical Education; Ministry of Education and Training, Child Growth and Development Research Center, Isfahan University of Medical Sciences; and Alborz University of Medical Sciences.

## Conflict of interest

The authors declare that they have no competing interests.

## Funding

This study was conducted as part of a national school-based survey.

## Highlights

- SES is one of the most important determinants of HRQOL in children.
- HRQOL score is higher in Iranian boys than girls.
- The association between SES and HRQOL in Iranian children and adolescents is mainly due to the impact of school functioning subscale.
